# First Report of *Enterocytozoon bieneusi*, *Encephalitozoon* spp. and *Blastocystis* spp. in Hair Goats in Türkiye

**DOI:** 10.1111/zph.70094

**Published:** 2026-09-15

**Authors:** Zuhal Önder, Sümmani Çifci, Batuhan Aşkım Arslanhan, Simge Şahin, Samba Deguene Diop, Alireza Sazmand, Önder Düzlü, Sadullah Uslu

**Affiliations:** ^1^ Department of Parasitology, Faculty of Veterinary Medicine Erciyes University Kayseri Turkey; ^2^ Department of Parasitology, Faculty of Veterinary Medicine Aksaray University Aksaray Turkey; ^3^ Department of Pathobiology, Faculty of Veterinary Medicine Bu‐Ali Sina University Hamedan Iran

**Keywords:** *Blastocystis* spp., *Encephalitozoon* spp., *Enterocytozoon bieneusi*, hair goats, molecular epidemiology, zoonotic importance

## Abstract

**Introduction:**

*Blastocystis* spp., *Enterocytozoon bieneusi* and *Encephalitozoon* spp. are prevalent zoonotic gastrointestinal parasites that cause severe diarrhoea and enteric diseases in humans and animals worldwide. This study investigated the molecular occurrence, genetic diversity and zoonotic potential of *E. bieneusi*, *Blastocystis* spp. and *Encephalitozoon* spp. in domestic hair goats in Central Anatolia.

**Methods:**

A total of 300 faecal samples were collected from hair goats in Kayseri, Aksaray and Sivas Provinces and genomic DNA was extracted from these samples. The presence of the three pathogens was detected using PCR and nested PCR targeting the SSU rRNA for *Blastocystis* spp., the internal transcribed spacer (ITS) for *E. bieneusi* and *Encephalitozoon* spp., respectively. Positive PCR products were sequenced to determine the species, subtypes and genotypes of the pathogens.

**Results:**

The overall prevalence rates of *E. bieneusi* and *Blastocystis* spp. were 5.3% (16/300) and 6.7% (20/300), respectively. None of the faecal samples tested was positive for *Encephalitozoon* spp. and no co‐infections among these three pathogens were detected. The SSU rRNA sequence analysis revealed *Blastocystis* spp. ST10, a subtype predominantly associated with animals. Additionally, one known *E. bieneusi* genotype (BEB6) was identified. The BEB6 genotype falls into zoonotic Group 2 of *E. bieneusi* in the phylogenetic tree.

**Conclusion:**

Our study is the first report of *Blastocystis* spp. and *E. bieneusi* infection in hair goats in Türkiye, highlighting their potential role in zoonotic transmission within a One Health framework. Our results provide scientific data for the prevention and control of these two intestinal pathogens. Further investigations are necessary to better understand their genetic characteristics and zoonotic potential in Türkiye.

## Impacts

- This study provides the first molecular evidence of *Blastocystis* spp. and *Enterocytozoon bieneusi* infections in native hair goats in Türkiye, addressing an important gap in knowledge of these parasites in small ruminants.
- The identification of *Blastocystis* subtype ST10 and *E. bieneusi* genotype BEB6, both reported in animals and humans, suggests a potential role of hair goats in their epidemiology and zoonotic transmission.
- These findings highlight the importance of a “One Health” approach, including improved farm hygiene and protection of local water sources, to minimize transmission risks among grazing livestock, farmers and surrounding communities.

## 1 Introduction

The livestock in Türkiye, especially small ruminant breeding, has a significant socio‐economic impact. Among these small ruminants, goats serve as an important source of livelihood for communities in rural and hard‐to‐reach areas (Hanoğlu Oral and Yıldız 2025). According to data from the Turkish Statistical Institute (TÜİK), Türkiye has one of the largest goat populations in Europe and the Mediterranean basin, with the native hair goat ( *Capra hircus* ) constituting nearly 98% of the national herd (TÜİK 2025). Hair goats are raised predominantly under extensive, pasture‐based traditional systems in rough and rocky terrains. These hardy animals provide a significant livelihood, supplying essential animal protein, meat, milk and leather to regional populations (Atac and Burcu 2014). However, the sustainability of these economically important hair goats is threatened by parasitic infections. Among these threats are *Blastocystis* spp., *Enterocytozoon bieneusi* and *Encephalitozoon* spp., which are opportunistic intestinal pathogens that can infect a wide range of hosts, including livestock, companion animals, wildlife and humans (Duan et al. 2024; Wang et al. 2022). These pathogens can be transmitted between humans and animals through varied routes, such as the consumption of food or water contaminated with spores or cysts and close contact with infected animals or humans (Hatam‐Nahavandi et al. 2025). Infections with these pathogens may cause severe abdominal pain, diarrhoea, weight loss, emaciation, lethargy and even death, posing significant public health risks and leading to considerable economic losses for the animal‐breeding industry (Hou et al. 2025; Li et al. 2022; Yu et al. 2023; Zhang et al. 2021).

The anaerobic unicellular eukaryote *Blastocystis* spp. is one of the most prevalent intestinal parasites in both humans and animals worldwide (Hou et al. 2025; Rajamanikam et al. 2023; Zhao et al. 2025). Globally, at least 44 subtypes (STs) of *Blastocystis* spp. have been identified in humans and animals based on sequence analysis of the small subunit ribosomal RNA (SSU rRNA) gene (Santin et al. 2024; Zhao et al. 2025). Host specificity is low among these subtypes and most subtypes have been reported in a variety of hosts (Hublin et al. 2021; Sazmand et al. 2019). Some of these subtypes (ST1 to ST10, ST12, ST14, ST16, ST23, ST35 and ST41) have been identified in both humans and animals, demonstrating the zoonotic potential of this parasite (Hublin et al. 2021; Popruk et al. 2021; Rehena et al. 2025). However, the pathogenic significance of *Blastocystis* spp. remains controversial and the dynamics of zoonotic transmission are not yet completely understood (Hou et al. 2025).

Microsporidia are obligate intracellular, spore‐forming unicellular eukaryotes that infect numerous invertebrate and vertebrate hosts, including humans. To date, more than 220 genera and 1700 species of microsporidia have been identified in various hosts (Han et al. 2021). The most common microsporidian infections in humans, domestic animals and wild animals are caused by *Enterocytozoon bieneusi* and *Encephalitozoon* spp. (Li, Feng, and Santin 2019; Li, Wang, and Gu 2019; Sazmand et al. 2024). Until now, more than 685 distinct genotypes of *E. bieneusi* in 236 different animal species (e.g., pigs, cats, dogs, horses and particularly cattle) and humans have been identified based on sequence analyses of the internal transcribed spacer (ITS) region of the rRNA gene and classified into 13 phylogenetic groups (Guadano‐Procesi et al. 2025; Li, Feng, and Santin 2019; Li, Wang, and Gu 2019; Qin, Liu, et al. 2022; Qin, Chen, et al. 2022). Among the genotype groups, Group 1 encompasses the major human‐pathogenic and zoonotic genotypes, including A, D, EbpC and Type IV. Group 2 has been determined to be host‐specific and mainly infects ruminants. Still, some of its members (such as genotypes I, J, BEB4 and BEB6) have also been reported in humans, suggesting that host specificity is not narrow (Duan et al. 2024; Jiang et al. 2023). Therefore, Group 2 genotypes pose potential risks to public health (Guadano‐Procesi et al. 2025; Koehler et al. 2022). The *Encephalitozoon* genus includes three species: *E. intestinalis* , *E. cuniculi* and *E. hellem*. Among these species, *E. cuniculi* is characterized by an extensive host spectrum, whereas *E. hellem* is predominantly limited to avian hosts. In contrast, *E. intestinalis* has been identified in wild animals only sporadically (Hinney et al. 2016).

In Türkiye, molecular research on these three opportunistic pathogens has focused on cattle, water buffaloes, sheep and camels from ruminants (Ayan, Aslan Celik, et al. 2024; Onder et al. 2021; Apaydın et al. 2023; Aynur et al. 2019; Öncü Öner et al. 2022; Tavur and Önder 2022; Ercan et al. 2024; Sursal Simsek et al. 2025). Consequently, a significant research gap remains regarding the status of these pathogens in hair goats; to the best of our knowledge, there are currently no published data on their prevalence, subtype distribution, or genotypic diversity. Given that these animals are raised under extensive pasture systems and remain in close contact with rural communities, understanding their epidemiological status is essential to uncovering previously overlooked zoonotic transmission pathways. Therefore, we aim to determine the molecular prevalence, genetic diversity and zoonotic potential of *Blastocystis* spp., *E. bieneusi* and *Encephalitozoon* spp. in hair goats in Türkiye. Determining the epidemiology and genetic diversity of these three pathogens in hair goat populations is essential for developing effective surveillance, prevention and control strategies and assessing their potential significance in zoonotic transmission pathways.

## 2 Materials and Methods

### 2.1 Ethical Approval

The present study is not subject to the permission of Local Animal Experiments Ethics Committees (HADYEK) in accordance with Article 8 (k) of the Regulation on Working Procedures and Principles of Animal Experiments Ethics.

### 2.2 Study Area and Collection of Faecal Specimens

A total of 300 faecal samples (100 from each province) from domestic hair goats, both male and female, of various ages and raised by local breeders, were collected across three provinces (Kayseri, Sivas and Aksaray) in the Central Anatolia Region of Türkiye, between May 2023 and July 2024 (Figure 1). Faecal samples were obtained from a total of 63 distinct smallholder farms across three provinces (21 farms in Kayseri, 26 in Sivas and 16 in Aksaray), with an average of 5 to 8 samples per farm to reach approximately 100 samples per province. All the sampled herds were raised under a traditional, semi‐extensive system. In this system, hair goats grazed freely in pastures during the day, accessing natural water sources. At night, they were kept in small, partially enclosed shelters and provided with safe, treated water. Furthermore, the removal of manure and faecal matter was regularly performed.

**FIGURE 1:**
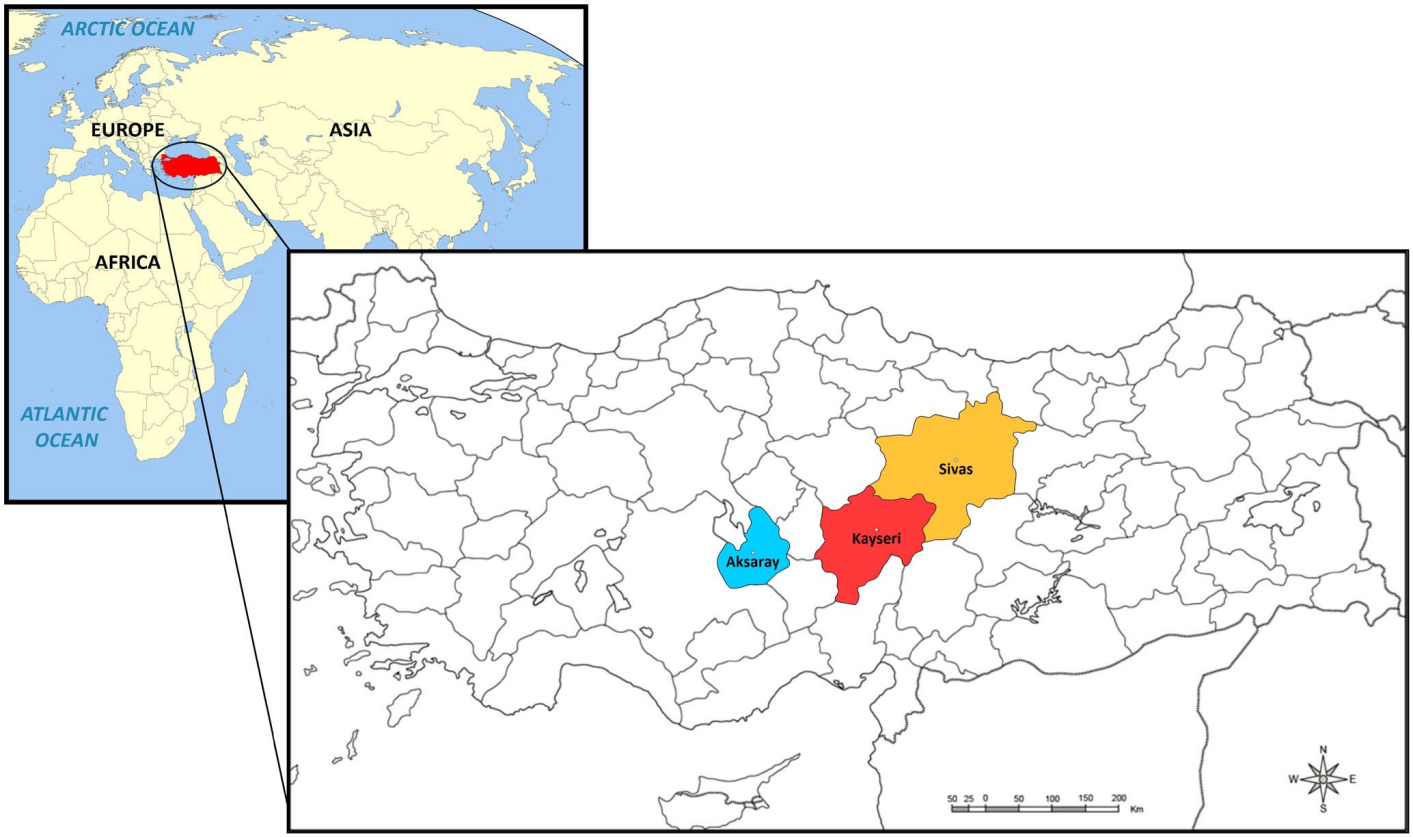
Map of sampling sites of hair goats in the Central Anatolia Region, Türkiye.

Fresh samples were collected from the upper portion of faecal deposits immediately post‐defecation to prevent soil contamination, using sterile disposable gloves and placed in individual plastic containers. All samples were transported to the laboratory under cold chain conditions. All hair goats involved in the study were clinically healthy and presented with normal faecal consistency at the time of sampling. Each goat's collection date, age, sex and province were recorded during the process. Samples were preserved at −20°C until genomic DNA extraction.

### 2.3 DNA Extraction and PCR Analyses

The genomic DNA from each faecal sample was extracted using the QIAamp DNA Stool Mini Kit (Qiagen, Hilden, Germany) in accordance with the manufacturer's protocol. The extracted genomic DNA was quantified using the Qubit dsDNA BR (Broad Range) Assay Kit (Thermo Fisher Scientific, Waltham, USA) to standardize the template concentration for PCR and stored at −20°C until PCR analysis.

To identify *E. bieneusi* genotypes and detect *Encephalitozoon* species, nested PCR assays targeting the internal transcribed spacer (ITS) region of the rRNA gene (~392 bp for *E. bieneusi* and ~305 bp for *Encephalitozoon* spp.) were performed as described by Buckholt et al. (2002) and Kasicková et al. (2009). *Blastocystis* spp. was screened using PCR amplification of a fragment (~600 bp) of the SSU rRNA gene, as described previously (Scicluna et al. 2006).

All PCR reactions were conducted in 25 μL reaction mixtures, comprising 2 μL of genomic DNA (for the primary PCR) or 1 μL of the first PCR amplification product (for the secondary PCR), 12.5 μL of DreamTaq Green PCR Master Mix (Thermo Fisher Scientific, Waltham, MA, USA; Cat. No. K1081), 1.25 μL of each primer (10 pmol/μL) and 8 μL of double‐distilled water. All amplifications were conducted using a C1000 Touch Thermal Cycler (Bio‐Rad, USA). Positive and negative controls were used in all the PCR tests performed. Finally, the PCR products were subjected to electrophoresis on a 1.5% agarose gel with SYBR Safe DNA (Invitrogen, USA) staining and visualized by the iBright CL1500 Gel Imaging System (Thermo Fisher Scientific, Waltham, MA, USA). Moreover, DNA extraction, PCR preparation and amplification were carried out in distinct work areas to prevent contamination. The primers and PCR conditions are summarized in Table 1.

**TABLE 1:** Primers and PCR conditions for identifying *Encephalitozoon* spp. and *E. bieneusi* by targeting the ITS gene region and *Blastocystis* spp. by targeting the SSU rRNA gene.

Pathogen	Gene	Primer name	Primer sequence (5′‐3′)	Amplicon size (bp)	Reaction condition	References
*Blastocystis* spp.	SSU rRNA	RD5	ATCTGGTTGATCCTGCCAGT	600	95°C for 3 min; 35 cycles of 95°C for 30 s, 56°C for 30 s and 72°C for 30 s; 72°C for 5 min	(Scicluna et al. 2006)
		BhRDr	GAGCTTTTTAACTGCAACAACG			
*Encephalitozoon* spp.	ITS	MSP 1	TGAATGKGTCCCTGT	305	95°C for 2 min; 35 cycles of 95°C for 30 s, 57°C for 30 s and 72°C for 1 min; 72°C for 7 min	(Kasicková et al. 2009)
		MSP 2A	TCACTCGCCGCTACT			
		MSP 3	GGAATTCACACCGCCCGTCRYTAT			
		MSP 4A	CCAAGCTTATGCTTAAGTYMAARGGGT			
*E. bieneusi*	ITS	EBITS3	GGTCATAGGGATGAAGAG	392	95°C for 2 min; 35 cycles of 95°C for 30 s, 55°C for 30 s and 72°C for 1 min; 72°C for 7 min	(Buckholt et al. 2002)
		EBTIS4	TTCGAGTTCTTTCGCGCTC			
		EBITS1	GCTCTGAATATCTATGGCT			
		EBITS2.4	ATCGCCGACGGATCCAAGTG			

### 2.4 Sequence and Phylogenetic Analysis

All amplicons exhibiting a single band of the expected size were cut from the agarose gel, purified using the GeneJet Gel Extraction Kit (Thermo Scientific, Waltham, USA) and sent to Macrogen Inc. (Macrogen, Amsterdam, Netherlands) for bidirectional sequencing. Primer sequences were removed from the sequencing reads. The raw sequence data from forward and reverse reads were processed, aligned and assembled using Geneious Prime 2025.0.3 software (https://www.geneious.com) to create final consensus sequences. The obtained final SSU rRNA gene sequences of *Blastocystis* spp. and ITS sequences of *E*. *bieneusi* were compared with reference sequences retrieved from the GenBank database using BLAST analysis (http://blast.ncbi.nlm.nih.gov/Blast.cgi). All nucleotide sequences identified in this study have been deposited in the GenBank Data Library under accession numbers PX678788‐PX678789 for *Blastocystis* spp. and PV226235‐PV226236 for *E. bieneusi*.

Phylogenetic trees derived from nucleotide sequences of *E. bieneusi* and *Blastocystis* spp. were constructed using the Maximum Likelihood (ML) method with 1000 bootstrap replicates in MEGA X software to assess their genetic relationships. The best‐fit nucleotide substitution model for each dataset was determined based on the lowest Akaike Information Criterion (AIC) score using jModeltest 2.1.7 (Darriba et al. 2012). Accordingly, the General Time Reversible model with gamma distribution and invariant sites (GTR + G + I) for the construction of the *Blastocystis* spp. tree (Figure 2) and the Hasegawa‐Kishino‐Yano model with gamma distribution and invariant sites (HKY + G + I) for the *E. bieneusi* tree (Figure 3) were selected. Only bootstrap values ≥ 50% were presented on the consensus trees.

**FIGURE 2:**
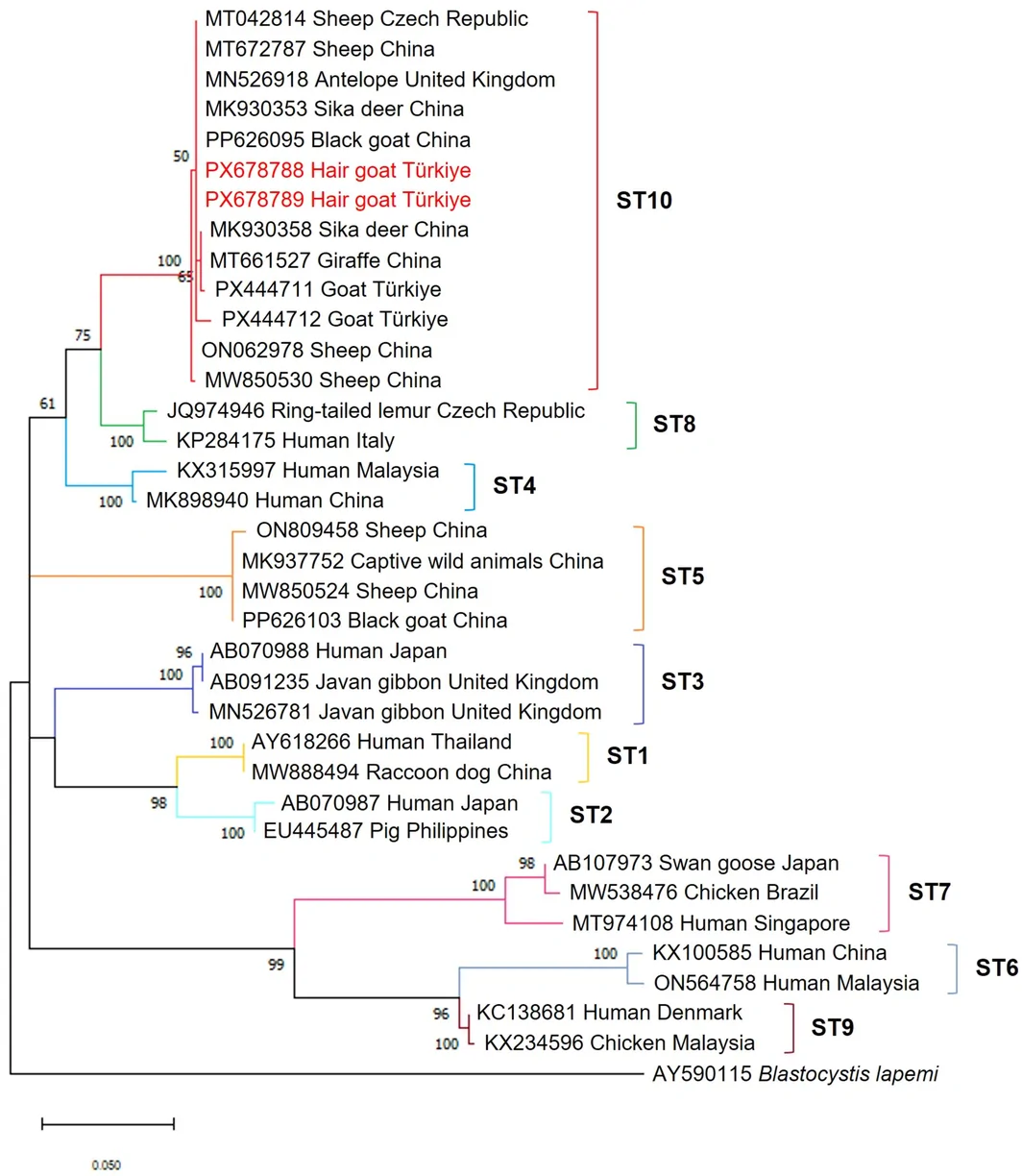
Phylogenetic tree of *Blastocystis* spp. isolates based on SSU rDNA gene sequences constructed using the Maximum Likelihood method in MEGA 11 software. The isolates identified in this study are indicated in red. Values on branches are per cent bootstrapping using 1000 replicates. GenBank accession numbers, host species and country of sequences are provided in the tree. The sequence of *Blastocystis lapemi* (AY590115) was used as the outgroup.

**FIGURE 3:**
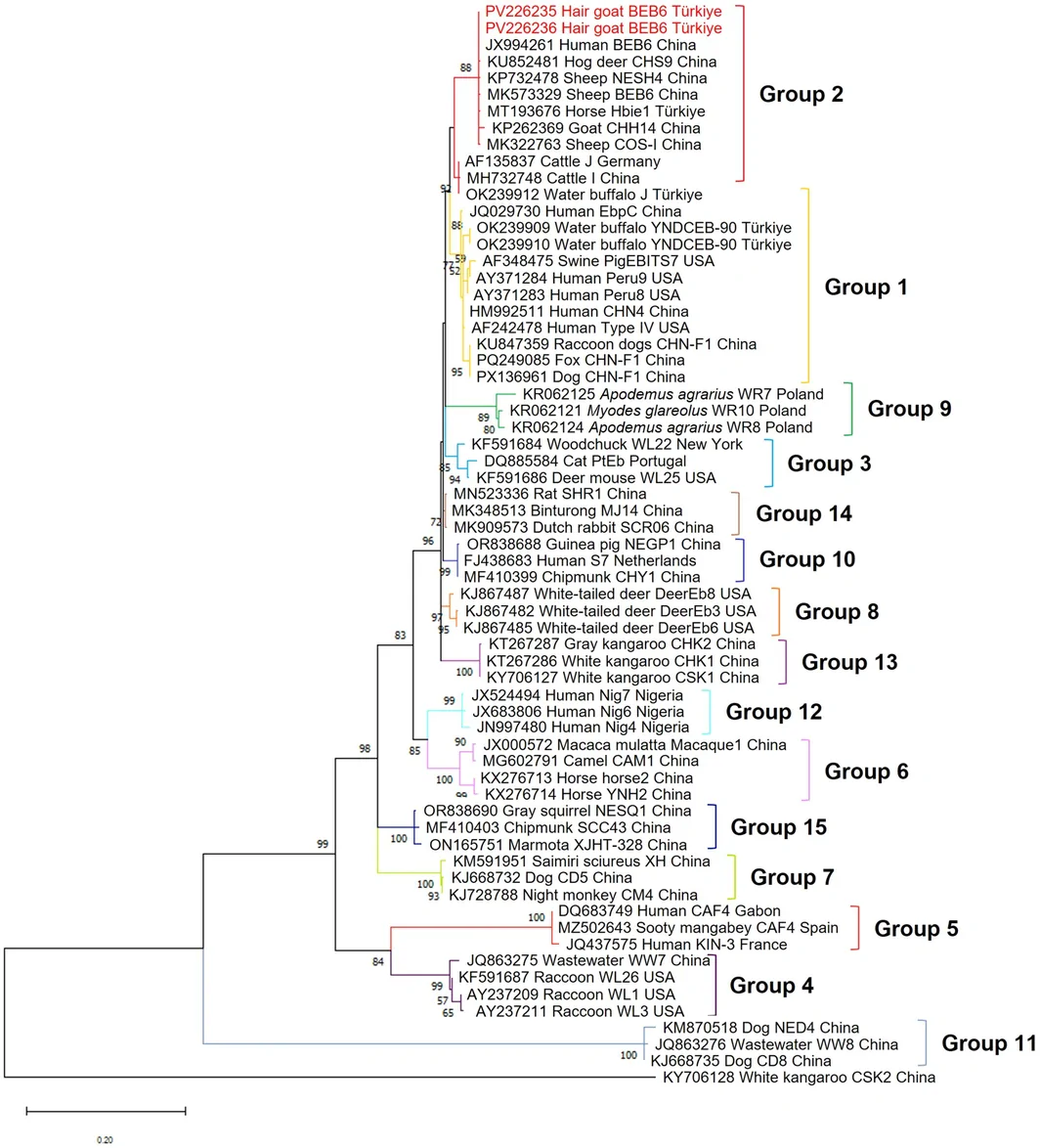
Phylogenetic analysis of *E. bieneusi* from hair goats inferred using the maximum likelihood method. Representative sequences obtained from this study are indicated in red. The numbers on the branches represent the bootstrap percentage values from 1000 repetitions. The isolates included in the data set are provided with their GenBank accession numbers, country, host name and corresponding genotype names. The *E. bieneusi* genotype CSK2 (KY706128) from the white kangaroo is used as the outgroup.

### 2.5 Statistical Analysis

All statistical analyses were performed using SPSS version 22.0 (SPSS Inc., Chicago, IL, USA). The prevalence of each parasite was calculated along with 95% confidence intervals (CIs) using the Wald method. Associations between infection status and categorical variables, such as province, sex and age group, were assessed using the chi‐square test. However, for subgroups where the expected frequencies fell below 5, Fisher's exact test was applied instead. The odds ratio (OR) with 95% CI was calculated to estimate the strength of associations. *p* values < 0.05 were considered statistically significant.

## 3 Results

### 3.1 Prevalence of *Blastocystis* spp., *E. bieneusi* and *Encephalitozoon* spp.

A total of 300 faecal samples collected from domestic hair goats in Kayseri, Sivas and Aksaray provinces were tested and 20 *Blastocystis* spp. positive samples were detected by PCR amplification of the SSU rRNA gene, representing an infection rate of 6.7% (95% CI: 3.9–9.5; Table 2). The highest prevalence was detected in Kayseri Province (10.0%, 95% CI: 4.1–15.9; 10/100), followed by Sivas Province (6.0%, 95% CI: 1.3–10.7; 6/100) and Aksaray Province (4.0%, 95% CI: 0.2–7.8). The prevalence did not differ significantly among the three provinces (*p* > 0.05). Regarding sex, the prevalence was 11.1% in males (95% CI: 4.3–18.0; 9/81) and 5.0% in females (95% CI: 2.1–12.6; 11/219). By age groups, the prevalence in goats younger than or equal to 1 year was 7.4% (95% CI: 2.1–12.6; 7/95), while in those older than 1 year, it was 6.3% (95% CI: 3.0–9.7; 13/205). Likewise, no statistically significant difference was observed (*p* = 0.740).

**TABLE 2:** Prevalence of *Blastocystis* spp. in hair goats.

Variable	No. of sample	No. of positive	Prevalence % (95% CI)	OR (95% CI)	*p*	Subtype
Province						ST10
Kayseri	100	10	10.0 (4.1–15.9)	1	0.223	
Aksaray	100	4	4.0 (0.2–7.8)	0.38 (0.11–1.24)		
Sivas	100	6	6.0 (1.3–10.7)	0.57 (0.20–1.65)		
Sex						
Female	219	11	5.0 (2.1–7.9)	1	0.061	
Male	81	9	11.1 (4.3–18.0)	2.36 (0.94–5.94)		
Age						
≤ 1 year	95	7	7.4 (2.1–12.6)	1.17 (0.45–3.05)	0.740	
> 1 year	205	13	6.3 (3.0–9.7)	1		
Total	300	20	6.7 (3.8–9.5)			

*Note: p*‐values were calculated using the Chi‐squared test and Fisher's exact test was applied for subgroups where the expected frequencies were less than 5.

Abbreviations: CI, Confidence Interval; OR, Odds Ratio.

Regarding *E. bieneusi*, a total of 16 positive samples were detected by nested PCR amplification of the ITS rRNA gene region, representing an infection rate of 5.3% (95% CI: 2.8–7.9; Table 3). The prevalence according to study area was 8.0% (95% CI: 2.7–13.3; 8/100) in Sivas Province, 6.0% (95% CI: 1.3–10.7; 6/100) in Kayseri Province and 2.0% (95% CI: 0.0–4.7; 2/100) in Aksaray Province, with no statistically significant difference among provinces (*p* > 0.05). By sex groups, males showed a higher prevalence (7.4%, 95% CI: 1.7–13.1; 6/81) compared to females (4.6%, 95% CI: 1.8–7.3; 10/219); however, the difference was not statistically significant (*p* = 0.331). By age groups, the prevalence in goats older than 1 year was 5.4% (95% CI: 2.3–8.5; 11/205), while in those younger than or equal to 1 year it was 5.3% (95% CI: 0.8–9.8; 5/95); the difference was not statistically significant (*p* = 0.971).

**TABLE 3:** Prevalence of *E. bieneusi* in hair goats.

Variable	No. of samples	No. of positive	Prevalence % (95% CI)	OR (95% CI)	*p*	Genotype
Province						BEB6
Kayseri	100	6	6.0 (1.3–10.7)	1	0.157	
Aksaray	100	2	2.0 (0.0–4.7)	0.32 (0.06–1.62)		
Sivas	100	8	8.0 (2.7–13.3)	1.36 (0.45–4.08)		
Sex						
Female	219	10	4.6 (1.8–7.3)	1	0.331	
Male	81	6	7.4 (1.7–13.1)	1.67 (0.59–4.76)		
Age						
≤ 1 year	95	5	5.3 (0.8–9.8)	0.98 (0.33–2.90)	0.971	
> 1 year	205	11	5.4 (2.3–8.5)	1		
Total	300	16	5.3 (2.8–7.9)			

*Note: p*‐values were calculated using the Chi‐squared test and Fisher's exact test was applied for subgroups where the expected frequencies were less than 5.

Abbreviations: CI, Confidence Interval; OR, Odds Ratio.

*Blastocystis* spp. and *E. bieneusi* were the most common species detected in the analysed hair goat samples. *Encephalitozoon* spp. were not detected among the samples.

### 3.2 Distribution of *Blastocystis* spp. Subtypes and *E. bieneusi* Genotypes

Our study identified one subtype of *Blastocystis* spp. and one genotype of *E. bieneusi* among the 300 hair goat samples. Based on SSU rRNA gene sequences, subtype ST10, which is predominantly associated with animals, was identified in all 20 *Blastocystis*‐positive samples. In the present study, 16 *E. bieneusi‐positive* samples were identified as the only known genotype BEB6 by comparative analysis of ITS gene sequences.

### 3.3 Phylogenetic Analysis

Phylogenetic trees were generated to represent the single *Blastocystis* spp. subtype (Figure 2) and the *E*. *bieneusi* genotype (Figure 3) identified in this study. Our ST10 sequences (PX678788‐89) showed 100% identity to isolates from sika deer in China (MK930353), antelope in the United Kingdom (MN526918), sheep in the Czech Republic (MT042814) and China (MT672787) and black goats in China (PP626095). The ML‐based phylogenetic analysis of the sequences acquired in this study revealed that the subtypes were distributed across distinct branches, with ST10 clustered alongside ST8.

According to the phylogenetic analysis of the ITS sequences obtained in our study and reference sequences retrieved from GenBank, the BEB6 genotype identified was classified within Group 2. Our BEB6 sequences (PV226235‐PV226236) exhibited 99.46% similarity to a sheep isolate from China (MK573329).

## 4 Discussion

*Blastocystis* spp. and *E. bieneusi* are among the most important zoonotic intestinal pathogens that cause significant losses in ruminants and constitute a potential threat to public health (Nourrisson et al. 2024; Yu et al. 2023). This study is the first molecular epidemiological survey in Türkiye investigating the presence, genetic diversity (subtypes and genotypes) and zoonotic potential of *Blastocystis* spp. and *E. bieneusi* in hair goats.

Regarding *Blastocystis* spp., the global prevalence of *Blastocystis* spp. in goats has been reported as 31.7% (837/2640), based on a previous systematic review (Rehena et al. 2025). Our study revealed the infection rate in hair goats was 6.7% (20/300). From a global perspective, the overall prevalence in the present study is lower than the rates reported in several Asian regions, such as Peninsular Malaysia (30.9%; Tan et al. 2013), Zhejiang Province (11.4%, 44/386; Zhao et al. 2025), Shaanxi Province (58.0%, 458/789; Song et al. 2017), Guangdong Province (33.63%, 76/226; Yu et al. 2023), Heilongjiang Province (50%, 7/14; Chen et al. 2023), Inner Mongolia (10.40%, Zhang et al. 2023) and the Qinghai‐Tibetan Plateau, China (8.46%, 22/260; Chang et al. 2021), western Thailand (94.74%, 36/38; Udonsom et al. 2018). Similar lower rates compared to our findings were also observed in Malaysia (38.0%, 19/50; Rauff‐Adedotun et al. 2022), Libya (10.5%, 4/38; Alfellani et al. 2013), Southwest France (14.2%, 1/7; Cian et al. 2017), the United States (75.0%, 6/8; Gould and Boorom 2013). However, the prevalence of *Blastocystis* spp. observed in this study is higher than that reported in Anhui Province, China (0.35%, 2/781, Li et al. 2018), Heilongjiang Province, China (4.8%, 8/166; Hou et al. 2025; 0.0%, 0/13; Wang et al. 2018), Shanxi Province, China (3.5%, 42/1200; Xiao et al. 2024), Nepal (3.4%, 1/29; Lee et al. 2012) and south‐eastern Iran (0.0%, 0/6; Hatam‐Nahavandi et al. 2025). The discrepancies in the prevalence of *Blastocystis* spp. among different studies may be attributable to numerous factors, including sample size, animal age, geographic location, feeding circumstances, climate, animal stocking density, management strategies and animal immunological status. In addition to these environmental and management factors, the wide variations in global prevalence may also be primarily related to methodological heterogeneity. Many previous studies have traditionally used conventional staining and microscopic evaluation. Although these classic diagnostic methods are practical, they have low diagnostic sensitivity and can frequently miss subclinical or low‐intensity infections where parasite shedding is minimal (Stensvold et al. 2007; Parkar et al. 2007). In contrast, the PCR assays used in our study target specific DNA sequences, exceeding the detection limits of conventional methods and preventing the potential for misidentification in morphological evaluations (Roberts et al. 2011). This highlights that the different prevalence rates reported worldwide are related not only to geographical variations but also to the choice of diagnostic method.

In our study, one subtype (ST10) of *Blastocystis* spp. was identified in hair goats in three provinces of Türkiye. These results are consistent with previous studies reported in Asia (Rauff‐Adedotun et al. 2022). Crucially, ST10 subtype was the most prevalent subtype of *Blastocystis* spp. in sheep, cattle and goats in Asia (Yu et al. 2023). This subtype has been reported in sheep, water buffalo and cattle in previous studies in Türkiye (Ayan, Aslan Celik, et al. 2024; Aynur et al. 2019; Öncü Öner et al. 2022; Onder et al. 2021; Tavur and Önder 2022). The ST10 subtype has also been reported in cats and dogs in Türkiye (Ayan, Aslan Çelik, et al. 2024). Importantly, the present study is the first to report the occurrence of *Blastocystis* ST10 in hair goats in Türkiye. Traditionally, ST10 is considered an animal‐specific subtype, predominantly found in various ruminants such as sheep, cattle and goats across Asia and Europe.

Specifically, the presence of ST10 in human populations has been reported in various countries, including Senegal (Khaled et al. 2020), Thailand (Jinatham et al. 2021), Vietnam (Nguyen et al. 2023) and Egypt (Naguib et al. 2023). This global distribution indicates that the subtype has a much broader host range than previously assumed, suggesting it may possess zoonotic potential under certain epidemiological conditions (Khaled et al. 2020; Wei et al. 2023). From a One Health perspective, which is important for assessing health risks that emerge at the intersection of human, animal and environmental health (Öz et al. 2025), identifying ST10 in hair goats in Türkiye is a significant finding.

Because this subtype can infect both livestock and humans globally, its presence in small ruminants suggests a hypothetical risk of zoonotic transmission in the study areas, whether through direct contact with infected animals or indirectly via faecal contamination of water and food. However, we must emphasize that direct evidence linking this animal isolate to human infections in Türkiye is currently lacking. Without concurrent local human clinical data or definitive transmission‐tracking studies, any active zoonotic dynamics in the study area remain speculative. Thus, while global data suggest we should reconsider the traditional view of ST10 as strictly ‘animal‐specific’, our findings highlight a potential vulnerability for people living close to livestock rather than evidence of a confirmed public health threat. Ultimately, these results highlight the need for integrated surveillance programs to monitor *Blastocystis* subtypes across humans, animals and their shared environments. Future research involving parallel human and animal sampling across broader geographical areas will be necessary to map precise transmission routes and determine impacts on local public health.

Globally, *E. bieneusi* has been recognized as a pathogen causing intestinal microsporidiosis in domestic small ruminants, posing a considerable public health concern due to its zoonotic potential (Gomes‐Gonçalves et al. 2023). However, limited data are available regarding *E. bieneusi* infections in goats globally. This study is the first report on the existence, prevalence (5.3%, 16/300) and genotypes of *E. bieneusi* in faecal samples collected from hair goats in three provinces in Türkiye. In this study, the prevalence of *E. bieneusi* in hair goats was lower than that reported in goats in Iran (18.5%, 37/200) (Hatam‐Nahavandi et al. 2025), Portugal (6.4%, 4/63) (Gomes‐Gonçalves et al. 2023), western Thailand (19.2%, 14/73) (Udonsom et al. 2019) and various provinces of China, including Heilongjiang (11.5%, 19/166, Hou et al. 2025; 21.8%, 12/55, Zhao et al. 2015), Tibet (9.6%, 25/260) (Chang et al. 2021), Yunnan (8.9%, 30/336) (Chen et al. 2018) and Jiangsu (35.54%, 209/588) (Cheng et al. 2024). However, it was higher than that in Slovakia (0.0%, 0/20) (Valenčáková and Danišová 2019) and other studies conducted in China, such as Shandong (0.0%, 0/24), Jiangsu (2.7%, 2/74), Henan (0.0%, 0/109) and Anhui (5.2%, 30/574) (Li, Feng, and Santin 2019; Li, Wang, and Gu 2019). Given these variations, extensive studies are needed to fully clarify the risk factors for *E. bieneusi* infection in goats, which depend on variables such as sample size, management system, diagnostic methods, farm hygiene and ecological conditions (Li, Feng, and Santin 2019; Li, Wang, and Gu 2019; Li et al. 2014).

To date, 49 *E. bieneusi* genotypes (44 discovered in China, with 12 being zoonotic) have been identified in goats. Among these, BEB6 and CHG3 are identified as the most common (Cheng et al. 2024). In the present study, one known *E. bieneusi* genotype (BEB6) was identified in hair goat populations. Many studies demonstrate that the genotype BEB6 was determined in goats in various provinces of China (Chen et al. 2018; Dong et al. 2022; Hou et al. 2025; Li, Feng, and Santin 2019; Li, Wang, and Gu 2019; Shi et al. 2016; Wang et al. 2022; Zhao et al. 2015) and Iran (Hatam‐Nahavandi et al. 2025). In Türkiye, *E. bieneusi* genotype BEB6 was previously identified in horses (Yildirim, Okur, et al. 2020), sheep (Apaydın et al. 2023), raw milk sampled from cattle and sheep (Yildirim, Al, et al. 2020), sheep milk and cattle milk cheese (Ercan 2025) and *Musca domestica* samples (Ercan et al. 2024). Phylogenetic analysis based on the ITS gene revealed that the BEB6 isolates obtained from different animal hosts clustered within zoonotic Group 2, which includes the genotypes most frequently found in ruminant hosts, but some of them have also been identified in humans, other animals and birds, such as BEB6 (Deng et al. 2019; Li, Feng, and Santin 2019; Li, Wang, and Gu 2019; Qin, Liu, et al. 2022; Qin, Chen, et al. 2022). Nevertheless, the host range for BEB6 is apparently broader than originally thought and the recent detection of this genotype in human hosts (Wang et al. 2013) indicates that this genotype might have zoonotic potential. However, human infections related to BEB6 remain exceptionally rare globally, with Wang et al. (2013) providing the only human case documented in the literature to date. This, to be less of data raises the question of whether an only report is epidemiologically sufficient to infer an actual public health risk in Türkiye. Given the complete absence of local human clinical data or direct transmission links in Türkiye, evaluating the actual regional risk posed by BEB6 remains highly challenging. Therefore, while its classification in Group 2 points to a potential for cross‐species transmission, our detection of BEB6 in hair goats should be interpreted with care. Rather than pointing to an active public health threat, this finding highlights the need for future multicenter epidemiological investigations involving simultaneous human and animal sampling to determine whether BEB6 has local zoonotic significance.

Data on the presence and prevalence of *Encephalitozoon* spp. in goats are limited worldwide, with reported seroprevalence rates of 12.5% in Denmark and 67.7% in Egypt (Cislakova et al. 2001; Abu‐Akkada et al. 2015). Indeed, a study conducted in the Czech Republic reported a prevalence rate of 3.3% in milk and faecal samples of goats by molecular methods (Vecková et al. 2021). In contrast, no *Encephalitozoon* infection was detected in hair goats examined in the current study. This result may be related to the limited sensitivity of conventional PCR in detecting low pathogen DNA loads, particularly in asymptomatic or immunocompetent animals. Therefore, future epidemiological studies should focus on utilizing more sensitive diagnostic methods such as quantitative PCR (qPCR) or serological tests (e.g., IFAT, ELISA), to accurately assess the true prevalence and status of *Encephalitozoon* infections.

Implementing comprehensive One Health control strategies is necessary to effectively reduce the risk of transmission of these pathogens. Farm measures should primarily focus on improving hygiene practices; in particular, the regular removal and proper composting of manure are crucial to minimize the environmental accumulation of spores and cysts (Van Herk et al. 2004). Furthermore, given that these pathogens are transmitted through water, routine monitoring of both communal animal watering troughs and nearby natural water sources is essential for early detection of faecal contamination (Karanis et al. 2007). Finally, comprehensive health screening programs should be established for occupationally exposed individuals such as local breeders, farm workers and veterinarians who manage these herds daily (Acke et al. 2022; Jokelainen et al. 2024). The integration of these agricultural, environmental and occupational health interventions provides a practical framework for preventing transmission pathways at the human‐animal‐environment interactions (Deiana et al. 2024).

The most important strength of this study is that it provides molecular epidemiological data and information on the genetic diversity (subtypes and genotypes) of *Blastocystis* spp. and *E. bieneusi* in hair goats in Türkiye, for which such information was previously unavailable. However, this study has certain limitations. First, the current study did not include other animal and human population surveys near these animals or environmental samples around the farms. Therefore, the potential transmission routes of these infections to humans could not be assessed from a One‐Health perspective. Second, the selection of farms in the study areas was based exclusively on owners' consent to participate and the convenience of accessing the animals for sampling. This limitation affects the generalizability of the results to the conditions prevalent in the three provinces of Türkiye. Third, since we sampled multiple animals within the same herd, a potential intra‐farm clustering effect could influence the statistical independence of our data and prevalence rates. We tried to minimize this effect by spreading our sample collection across 63 different smallholder farms rather than focusing on just a few farms. Fourth, clinical signs were not recorded, and detailed faecal consistency scoring was not performed, beyond confirming that the sampled animals appeared healthy and produced ‘normal’ faecal samples at the time of collection. Thus, no associations with clinical disease or pathogenicity could be established. Fifth, collecting a single faecal sample from each animal presents a limitation, as it may fail to detect parasites due to their intermittent shedding in faeces, potentially leading to an underestimation of the actual prevalence. Despite these limitations, the present study successfully confirmed the occurrence of *Blastocystis* spp. and *E. bieneusi* in hair goats and provided valuable preliminary data into their genetic diversity, including subtypes and genotypes.

## 5 Conclusion

Our study is the first to examine the molecular epidemiology and genotype distribution of *Blastocystis* spp., *Encephalitozoon* spp. and *E. bieneusi* in the hair goat populations in Türkiye. The detection of the zoonotic *E. bieneusi* genotype BEB6 and predominantly animal‐specific *Blastocystis* subtype 10 in the hair goats emphasizes a potential public health risk through close contact with infected animals or their faeces, or via contamination of groundwater and surface water. Consequently, further detailed research should be carried out in various regions of Türkiye to better understand the molecular epidemiology, genetic traits, zoonotic potential and transmission routes of these intestinal pathogens.

## Funding

The Erciyes University Research Fund financially supported this research (TSA‐2023‐12941).

## Conflicts of Interest

The authors declare no conflicts of interest.

## Data Availability

The data that support the findings of this study are available on request from the corresponding author. The data are not publicly available due to privacy or ethical restrictions.
